# Pinocembrin suppresses proliferation and enhances apoptosis in lung cancer cells in vitro by restraining autophagy

**DOI:** 10.1080/21655979.2021.1972779

**Published:** 2021-09-04

**Authors:** Hongxia Gong

**Affiliations:** Traditional Chinese Medicine Department, Yuhuangding Hospital, Yantai, Shandong Province, China

**Keywords:** Pinocembrin, autophagy, lung cancer, proliferation, apoptosis, caspase3

## Abstract

Lung cancer is one of the leading causes of human death, and the 5-year survival rate for lung cancer patients remains a relative low level. Pinocembrin (Pino) was reported to play an important role in the inhibition of cancer development, so this study was designed to explore the role of pino in lung cancer. A549 cells were treated with different concentration of Pino (25, 50, 100, 150 and 200 µM) for 24, 48 and 72, respectively to detect cell viability by Cell counting kit-8 (CCK-8) assay. Then, the proliferation, apoptosis and autophagy of A549 cells under pino exposure were detected using colony formation, TUNEL and immunofluorescence staining, respectively. Western blot was used to analyze proliferation-, apoptosis-, and autophagy-related proteins. To measure the effects of pino on cell autophagy, the above-mentioned functional assays were conducted again in A549 cells treated with pino and 20 µM autophagy activator rapamycin (RAPA). Declined trends in cell viability, proliferation, and autophagy were found in A549 cells treated with increasing doses of pino, by contrast with those without any treatment. Additionally, the apoptosis of A549 cells was enhanced upon pino exposure, accompanied by elevated caspase3 activity. However, RAPA reversed the anti-proliferative, anti-autophagic and pro-apoptotic properties of pino in A549 cells. In conclusion, this paper is the first to verify that pino suppresses the proliferation and enhances the apoptosis of lung cancer cells by restraining autophagy, indicating that pino has potential therapeutic effects on the treatment of lung cancer.

## Introduction

Lung cancer is one of the leading causes of human death, and the 5-year survival rate for lung cancer patients remains a relative low level [1]. Patients with lung cancer are manifested with nonspecific symptoms, such as fatigue, anorexia, and weight loss, which are the main causes for missing the optimal diagnosis time [2]. Although the diagnosis and treatment of lung cancer have progressed recently, the recurrence of lung cancer is still high [3]. Therefore, identifying novel treatment methods is of great urgency for the suppression of lung cancer progression.

Pinocembrin (5,7-dihydroxyflavone; Pino) can be extracted from aerial parts of *Flourensia oolepis S.F. Blake (Asteraceae)* and honey [4,5]. As a flavonoid natural compound, it can be found in several fruits, vegetables, nuts, seeds, herbs, spices, stems, flowers, tea, and red wine [6]. Apart from the previously reported antimicrobial, anti-inflammatory and antioxidant effects, pino also plays a neuroprotective role in cerebral ischemic injury [7] and serves as a superb candidate for anti-allergic therapies [8,9]. A large number of studies have evidenced that pino is beneficial for the treatment of cancers. The proliferation and migration of ovarian cancer cells can be suppressed by pino via decreasing the expression of N-cadherin and gamma-aminobutyric acid receptor [10]. Pino treatment led to decreased proliferation, migration, and invasiveness of colorectal cancer cells [10]. The ability of pino to suppress phosphatidylinositol 3 kinase /Akt/ mammalian target of rapamycin pathway and trigger apoptosis of melanoma cells indicates the therapeutic potential of pino for the treatment of melanoma [11].

Autophagy is a tightly orchestrated cellular degradation and recycling process [12]. It can be classified into three major steps, which are microautophagy, macroautophagy, and chaperone-mediated autophagy (CMA) [12]. The recycling process of autophagy is essential for the homeostasis of both physiological and pathophysiological events [13]. Once the autophagy ability in cells is destroyed, a variety of diseases including neurodegenerative diseases, infectious disease, type II diabetes, and cancer will occur [14,15]. Research has shown that pino can induce ER stress-modulated apoptosis and refrain autophagy in melanoma cells, implicating that pino participates in the regulation of autophagy [11]. Thus, we predicted that Pin could have cytotoxicity effects on lung cancer cells, which could be related to the induction of proliferation inhibition, apoptosis and decreased autophagy.

In the present study, we probed into the role of pino in lung cancer and its possible relationship with autophagy, thereby revealing that the role of pino in lung cancer cells and its action mechanism involved in apoptosis, proliferation and autophagy.

## Materials and methods

Cell culture and drug administration

Human lung cancer cells A549 was provided by Type Culture Collection of the Chinese Academy of Sciences (Shanghai, China). Cells were cultured in DMEM supplemented with 10% heat-inactivated fetal bovine serum (FBS, Gibico) in a humidified atmosphere with 5% CO_2_ at 37 C. Pino was provided by Yuanye Bio-Technology (Shanghai, China). When cells grew to 80% confluence, Pino treatment was conducted for the following experiments, together with treatment with 20 µM autophagy activator rapamycin (RAPA; Sigma-Aldrich; Merck KGaA).

CCK-8 assay

Cells were inoculated in 96-well plates and treated with different doses of pino (25, 50, 100, 150, and 200 µm) for 24, 48, and 72 h. The choose of concentration gradients and treatment time of Pino was based on preliminary experiment and other previous study [10]. After treatment with 10 μL CCK-8 solution (MedChemExpress, Shanghai), the cells were incubated for another 4 h at 37°C in an atmosphere of humidified containing 5% CO_2_. The absorbance at 450 nm was evaluated using a spectrophotometer (Thermo Fisher Scientific, Camarillo, CA, USA).

Immunofluorescence staining

After cells were treated with different concentrations of pino for 72 h, immunofluorescence staining was performed. The slides were incubated at 4°C overnight with the primary antibody against LC3 (GB13431, Servicebio, China). On the next day, the slides were washed by PBS for twice and then incubated with fluorescence-conjugated secondary antibody (1: 200, Jackson ImmunoResearch, USA) at room temperature for 2 h. Following that, DAPI staining was carried out to dye the nuclei in the cells, and the images were captured for the analysis of the fluorescence intensity by ImageJ software.

## Colony formation assay

A549 cells pretreated with pino were inoculated into 96-well plates at the density of 2 × 10^4^ cells/well and incubated for 48 h. After washing with PBS for three times, the cells were fixed with 4% methanol, and stained with crystal violet. The photographs of colonies were visualized under a microscope to evaluate the colony number.

## Western blot

After extracting the proteins from A549 cells in the RIPA lysis buffer, the protein samples were isolated by sodium dodecyl sulfate-polyacrylamide gel electrophoresis (SDS-PAGE), and transferred to polyvinylidene difluoride (PVDF) membranes (Millipore, Billerica, MA, USA). The membranes were incubated with primary antibodies overnight at 4 C, and subsequently incubated with goat anti-rabbit IgG for 1 h. The images of proteins were obtained using enhanced chemiluminescence kit (BL520B, Biosharp, China). The analysis of the relative band intensity was carried out by Quantity One software.

Terminal deoxynucleotidyl transferase-mediated dUTP nick-end labeling (TUNEL) Prior to the transference to the slides, cells were treated with various concentrations of pino or 0.1% DMSO for 48 h. Afterward, cells were fixed and permeabilized, washed with PBS twice and then detected by TUNEL detection kit (Roche Diagnostics, Indianapolis, IN, USA) according to the manufacturer’s instructions. The pictures of apoptotic cells were obtained by microscopy (Olympus BX51, Japan).

Flow cytometry

A549 cells were collected, digested with trypsin and resuspended with PBS. Subsequently, cells were mixed with Binding Buffer 500 μL. The apoptosis levels of cells were detected with ANNEXIN V- FITC/PI kit (Solarbio LIFE SCIENCES, Beijing, China) according to manufacturer’s protocol. AnnexinV-FITC 5 µL was added into the mixture, followed by the addition of PI 5 μL to incubate with cells in room temperature in dark. The apoptosis levels were analyzed under CytoFLEX flow cytometry (ABI, USA).

Detection of caspase3

The activity of caspase3 was measured by Caspase-3 Activity Assay Kit (Sigma-Aldrich, Germany) in accordance with the manufacturer’s recommendations. Briefly, cells were digested with typsin and centrifuged at 600 g for 5 min at 4°C. Then, cells were lysed with Lysis buffer for 15 min at ice and then centrifuged at 2000 g for 10 min to collect supernatant. Following the addition of Ac-DEVD-pNA, the absorbance was detected at 405 nm.

## Statistical analysis

All results are expressed as the mean ± standard deviation (SD). Data were analyzed on the GraphPad Prism software (GraphPad Software Inc., San Diego, CA, USA). Student’s t-test was used for the comparison between two groups, and one-way ANOVA analysis followed by Tukey’s multiple comparisons test was used for the comparisons among multiple groups. Value of P < 0.05 was considered to be statistically significant.

## Results

Pino decreased the cell viability of A549 cells

**Figure 1.**
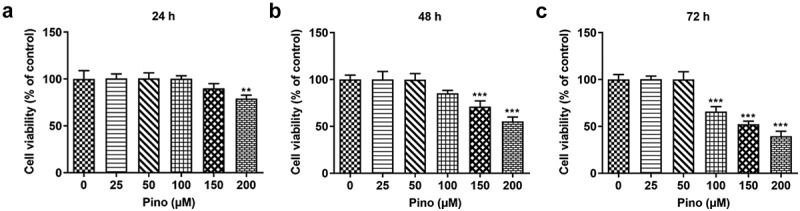
Pino decreased the cell viability of A549 cells. Cell viability was detected using CCK-8 assay at (a) 24 h, (b) 48 h and (c) 72 h. **P < 0.01, ***P < 0.001 versus Pino 0 µM

To determine the role of pino in lung cancer, we first examined the cytotoxic influence of pino on A549 cells. As shown in Figure 1a, the cell viability of A549 cells treated with 25, 50 and 100 µm pino for 24 h stayed unchanged, while 150 and 200 µm pino slightly decreased the cell viability. As Figure 1(b-c) displayed, pino with concentrations of 100, 150 and 200 µm greatly decreased the cell viability of A549 cells t. When we extended the treatment time of pino to 48 h or 72 h, the cell viability of A549 cells exposed to 100, 150 and 200 µm pino descended remarkably compared with those treated with pino at the concentrations of 0, 25, and 50 µm. Therefore, we chose 100, 150 and 200 µm pino for the following experiments.

Pino decreased the proliferation and promoted the apoptosis of A549 cells

**Figure 2.**
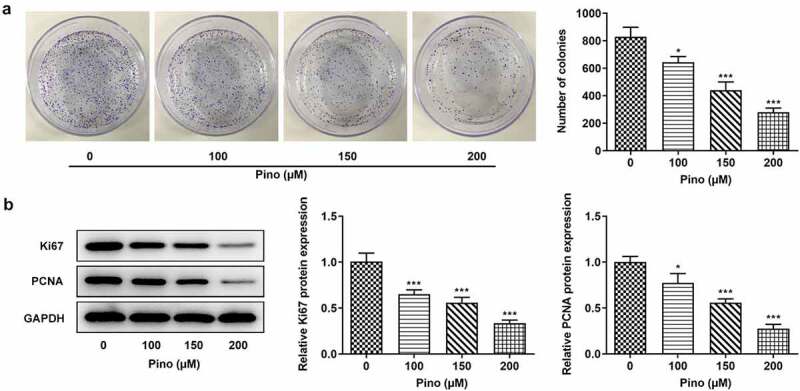
Pino decreased the proliferation of A549 cells. (a) The colony formation of A549 cells upon exposure to various concentrations of pino. (b) The expression of proliferation-related proteins in A549 cells after pino exposure. *P < 0.05, ***P < 0.001 versus Pino 0 µM

**Figure 3.**
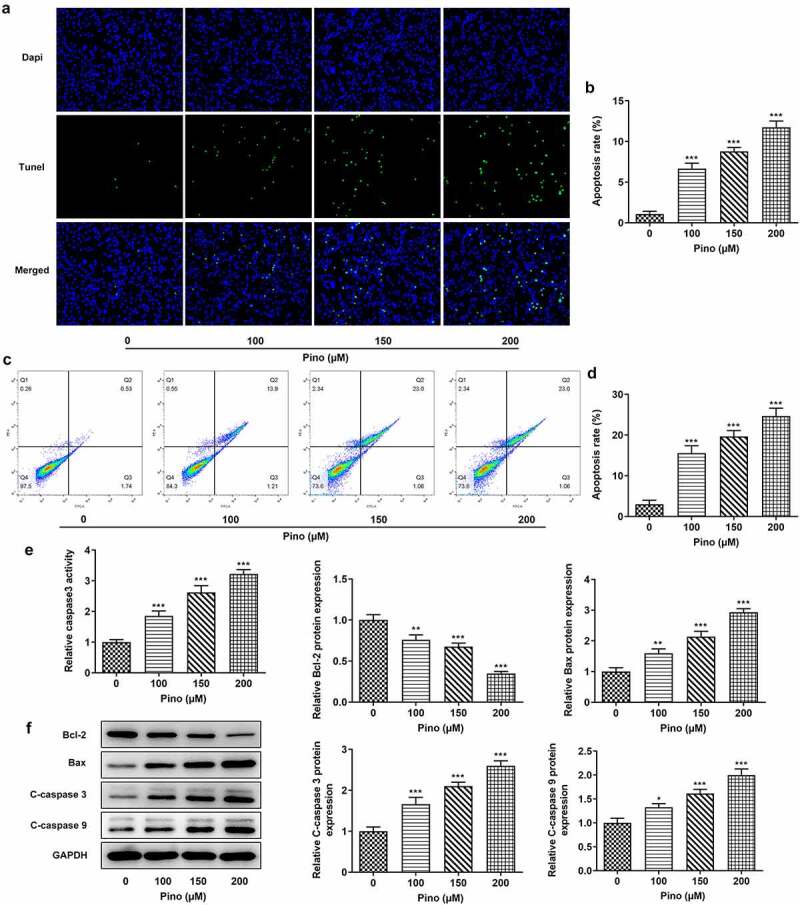
Pino promoted the apoptosis of A549 cells. (a, b) The apoptosis of A549 cells upon exposure to various concentrations of pino. (c) The activity of caspase3 detected by corresponding commercial kits in A549 cells exposed to pino. (d) The apoptosis-related protein levels were examined after A549 cells treated with pino. *P < 0.05, **P < 0.01, ***P < 0.001 versus Pino 0 µM

Then, we further explored the effects of Pino on proliferation and apoptosis. The effects of pino on A549 cells proliferation was detected using colony formation assay. As exhibited in Figure 2a, the number of colonies was significantly diminished after the exposure to pino. Moreover, the inhibitory effects of pino on A549 cells proliferation were in a concentration-dependent manner. Concurrently, the expression of proliferation-related markers, which are Ki67 and PCNA, decreased with the increase of pino dose in A549 cells (Figure 2b). Following that, the effects of pino on the apoptosis of A549 cells were detected using TUNEL. Notably, pino greatly increased the apoptosis rate of A549 cells as well as the activity of caspase3 (Figure 3a-c). Additionally, the relative protein expressions of Bax, cleaved caspase3 and cleaved caspase9 were significantly upregulated by pino, while Bcl-2 protein expression was hugely downregulated (Figure 3d). Taken together, pino decreased the proliferation and promoted the apoptosis of A549 cells.

Pino reduced the autophagy of A549 cells

**Figure 4.**
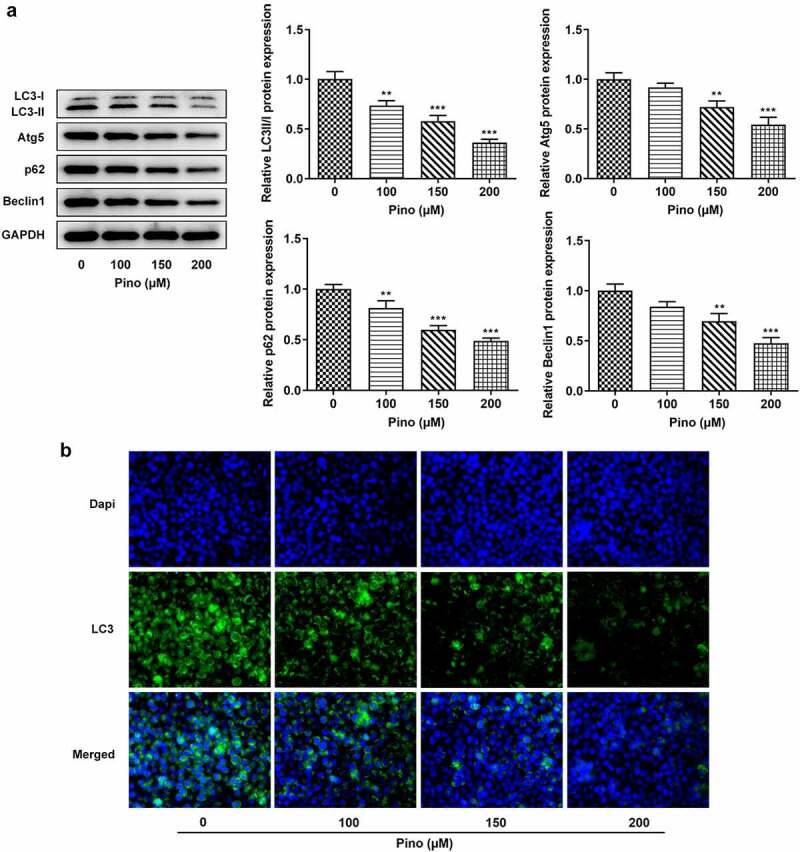
Pino reduced the autophagy of A549 cells. (a) The autophagy protein levels in A549 cells treated with pino were examined by western blot . (b) The autophagy level in pino-treated A549 cells was measured by immunofluorescence staining. **P < 0.01, ***P < 0.001 versus Pino 0 µM

Afterward, whether pino could affect the autophagy of A549 cells was investigated. The expressions of autophagy proteins were detected using western blot, and the results in Figure 4a showed that the expressions of LC3-II, Atg5, P62, and Beclin1 were downregulated by pino in a concentration-dependent manner. Meanwhile, immunofluorescence staining of LC3 displayed that the effect of pino on autophagy was in a dose-dependent manner (Figure 4b). These results suggested that pino reduced the autophagy of A549 cells.

RAPA reversed the suppression of pino on the autophagy and proliferation of A549 cells

**Figure 5.**
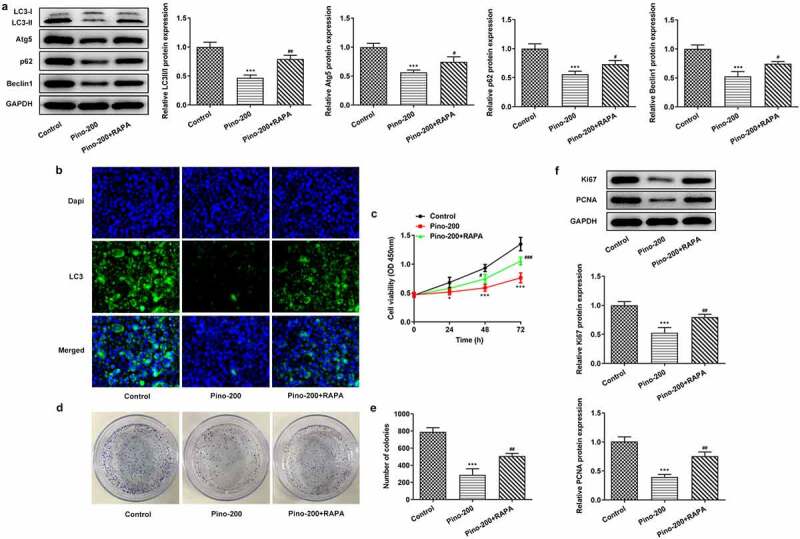
RAPA reversed the suppression of pino on the autophagy and proliferation of A549 cells. (a-b) The expression of autophagy proteins, (c) cell viability, (d, e) colony formation, and (f) expression of proliferation-related proteins were examined in A549 cells co-treated with pino and RAPA. ***P < 0.001 versus Control, *^#^P < *0.05, *^##^P < *0.01 versus Pino-200

To confirm whether pino exerted inhibitory effects on the proliferation of A549 cells by restraining autophagy, we used 20 µM autophagy activator RAPA to treat A549 cells. The expression of autophagy proteins was reduced remarkably upon exposure to 150 µm pino, which was reversed by RAPA (Figure 5a-b). Furthermore, the cell viability of A549 cells decreased by pino was partially restored by RAPA treatment (Figure 5c). Compared with Control, pino treatment markedly reduced the number of colonies in A549 cells, which was alleviated by co-treatment with pino and RAPA (Figure 5d-e). Similarly, the inhibited expression of proliferation-related markers was greatly increased by the treatment of RAPA. (figure 5f). Therefore, RAPA reversed the inhibitory effects of pino on the autophagy and proliferation of A549 cells.

RAPA reversed the promotive effects of pino on the apoptosis of A549 cells

**Figure 6.**
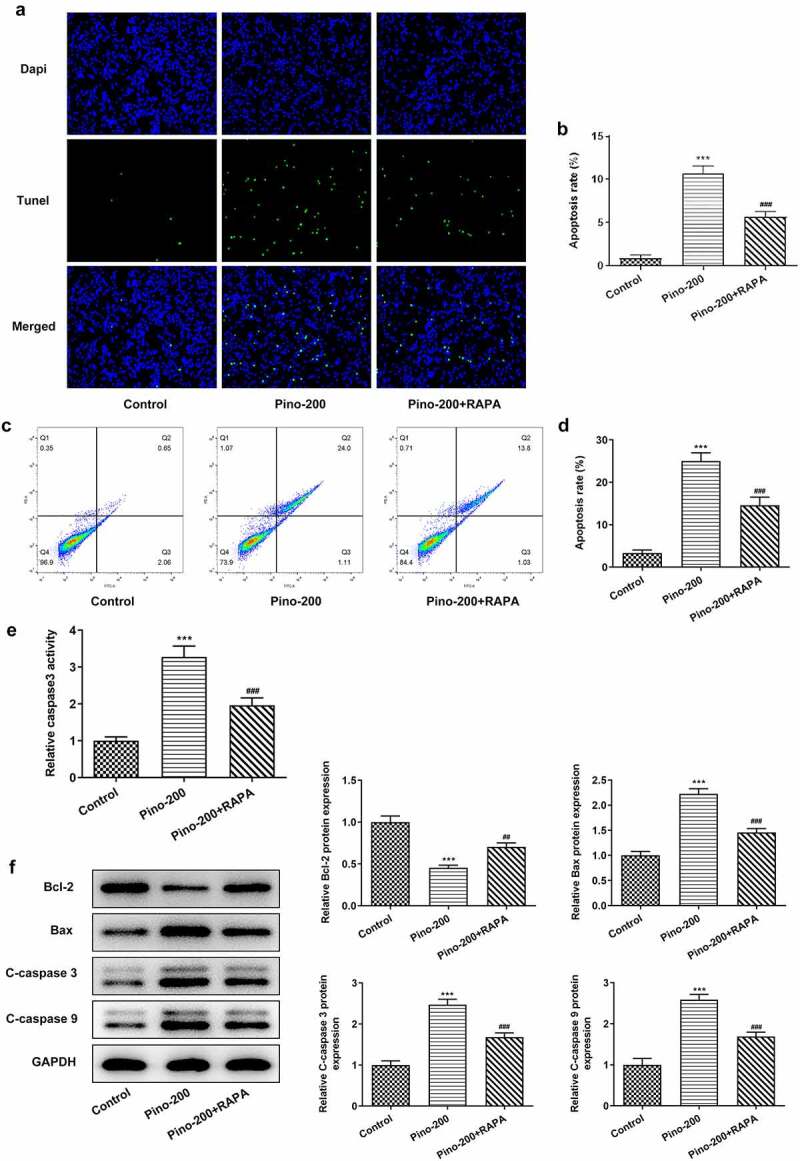
RAPA reversed the promotive effects of pino on the apoptosis of A549 cells. (a) The apoptosis, (b) caspase3 activity and (c) apoptosis-related proteins in A549 cells exposed to pino and RAPA. ***P < 0.001 versus Control, *^##^P < *0.01, *^###^P < *0.001 versus Pino-200

Then, RAPA was used to treat A549 cells to evaluate whether pino promoted the apoptosis of A549 cells by restraining autophagy. TUNEL analysis demonstrated that pino greatly increased the apoptosis rate of A549 cells, which was reversed by RAPA (Figure 6a,b). Interestingly, the activity of caspase3 was increased in pino-treated A549 cells whereas the adding of RAPA partially decreased that increased activity (Figure 6c). Finally, decreased expression of Bcl-2 together with elevated expressions of Bax, C-cleaved3 and C-cleaved9 was found in A549 cells treated with pino (Figure 6d). Nonetheless, this phenomenon was reversed after co-treatment with pino and RAPA compared with A549 cells treated with only pino. These results indicated that RAPA reversed the promotive effects of pino on the apoptosis of A549 cells.

## Discussion

Pino can be extracted from tree buds or exudates of plants, and its use can be traced back to ancient times due to its pharmacological effects [16]. It has been previously reported that pino can treat neuroblastoma, ischemia reperfusion and cancers [17]. However, there is no study reporting the role of pino in lung cancer development. In this study, we first detected the cytotoxic effects of pino on A549 cells, finding that pino with concentrations of 100, 150, and 200 µm hugely decreased the cell viability and proliferation of A549 cells. Treatment with pino for 24 h could only slightly affect the cell viability of A549 cells, while treatment with pino for 48 h drastically decreased the cell viability, especially at the concentrations of 100, 150 and 200 µm, suggesting the beneficial role of pino against lung cancer cell proliferation. Lacking of the evaluation for the effects of Pino on malignant biological behavior of A549 cells invasion and metastasis is the limit of this study and requires further deeper study. Substantial studies have reported the critical role of pino in mediating cell apoptosis, and we also observed the effects of pino on cell death [18]. Herein, the apoptosis of A549 cells was dramatically promoted with the increasing doses of pino. The death of most cells depends on the caspase3 pathway, meanwhile, the increased activity of caspase3 was also found in A549 cells treated with pino [18]. Autophagy also has great potential to mediate cell death, which makes it be a potential target for the treatment of various cancers [19–23]. Thus, we speculated that pino might induce the apoptosis of A549 cells via the regulation of autophagy.

The past few decades have witnessed the progress that has been made in targeting autophagy for the treatment of cancer [13]. Autophagy starts with a phagophore possibly originated from lipid bilayer, which is able to gobble up the wastes in the process of cell metabolism, including protein aggregates, organelles and ribosomes [24]. A previous study evidenced that the inhibition of autophagy might play a prominent role in cancer treatment [25]. Defective autophagy can indirectly influence the tumor microenvironment, stimulate oxidative stress and raise the possibility of cancer-related mutations [26,27]. Deficiency of autophagy genes can also boost the necrosis and inflammation which are critical contributors to multiple cancers, and the findings of previous studies demonstrated that autophagy can affect the tumor growth in lung cancer, pancreatic cancer and melanoma [26,28]. Pino has been verified to be related with autophagy in various diseases. Pino can alleviate the damage in pyramidal neurons of ischemia/reperfusion rat model by suppressing the excessive activation of autophagy, thereby protecting the rats from brain injury [7]. Consistent with the findings of previous studies, the expressions of autophagy proteins in A549 cells were reduced by pino, demonstrating the possible relationship between autophagy and pino in lung cancer cells. In addition, the suppressed proliferation in A549 cells upon pino exposure were restored by RAPA treatment while the promoted apoptosis was inhibited. Pino might represent a natural agent used for the therapies of glucocorticoid-induced osteoporosis (GIOP) as it was reported to ameliorate glucocorticoid-induced osteocyte apoptosis by activating autophagy via the inhibition of PI3K/Akt/mTOR pathway [29]. Besides, Reactive Oxygen Species-involved PI3K/AKT/mTOR and ERK/p38 MAPK signaling mediated apoptosis by autophagy inhibition in human prostate cancer cells [30]. These provided a sight for explaining how decreased autophagy by pino mediated cell apoptosis. Taken together, these results indicated that the inhibition of autophagy by Pino could suppress A549 cells proliferation and promote apoptosis.

## Conclusion

This paper is the first to testify that pino suppressed the proliferation and promoted the apoptosis of lung cancer cells by restraining autophagy, indicating that pino has potential therapeutic effects on the treatment of lung cancer. However, there are some limitations in the present study, such as the lack of an *in vivo* model to verify the practical effects of pino.

## Highlights

1. Pinocembrin has potential anti-tumor effects through suppressing cell proliferation and promote apoptosis in lung cancer.

2. Autophagy participates in the anti-tumor growth of pinocembrin.

3. The suppression of cell proliferation and the promotion of apoptosis by pinocembrin partly depending on autophagy suppression.
